# Idiopathic lung fibrosis and anti myeloperoxidase glomerulonephritis: the tree that hides the forest

**DOI:** 10.1186/s12890-015-0129-5

**Published:** 2015-10-26

**Authors:** Marc Pineton de Chambrun, Hilario Nunes, Isabelle Brochériou, Alexandre Hertig

**Affiliations:** APHP, Hôpital Tenon, Urgences Néphrologiques et Transplantation Rénale, Hôpital Tenon, 4 rue de la Chine, 75020 Paris, France; APHP, Hôpital d’Avicenne, Service de Pneumologie, Bobigny, France; Université Paris 13, Sorbonne Paris Cité, EA2363 “Réponses cellulaires et fonctionnelles à l’hypoxie”, Paris, France; APHP, Hôpital Tenon, Anatomopathologie, Paris, France; UPMC Sorbonne Université Paris 06, UMR S 1155, F-75020, Paris, France

**Keywords:** Idiopathic pulmonary fibrosis, Anti-Neutrophil Cytoplasmic Antibody Associated Vasculitis

## Abstract

**Background:**

Although anti-neutrophil cytoplasmic antibodies [ANCA] are frequently found in patients diagnosed with idiopathic pulmonary fibrosis [IPF], current guidance does not recommend serologic testing for vasculitis.

**Case presentation:**

A 71-year old Caucasian male, diagnosed with IPF three years earlier, presented with rapidly progressive glomerulonephritis. ANCA were found both in current and historical sera. A kidney biopsy sample was taken, which revealed a pauci-immune glomerulonephritis, but also areas of glomerular fibrosis, hence strongly suggesting unrecognized flares of an indolent vasculitis in his past. This made the diagnosis of “idiopathic” pulmonary fibrosis very unlikely.

**Conclusion:**

As nephrologists, we argue that testing for ANCA should be performed on a systematic basis, at least in elderly patients, even in the absence of extra-pulmonary signs of vasculitis at presentation.

## Background

Idiopathic pulmonary fibrosis [IPF] is a progressive fibrotic disorder of the lower respiratory tract with increasing incidence rates [1]. By definition, to reach a diagnosis of IPF other possible causes of interstitial lung disease, such as domestic or occupational environmental exposure, drug toxicity, and connective tissue diseases must be excluded [2]. For the latter, a worldwide consensus was reached recently that a serologic evaluation should be systematically recommended, particularly for younger women; this would include testing for rheumatoid factor and anti-cyclic citrullinated peptide, and carrying out an antinuclear antibody (titer and pattern) on the basis of a high benefit/cost ratio [2]. Serologic testing for vasculitis is not included in this guidance [2]. Mild pulmonary hemorrhage may complicate antineutrophil cytoplasmic antibody [ANCA]-associated vasculitis [AAV] and, more importantly, it may occasionally go unnoticed. Since fibrosis is a non-specific lesion resulting from a perpetuating or failing repair process that is independent of the cause of injury, it is reasonable to think that mild pulmonary flares might cause interstitial lung disease. As a matter of fact, Japanese and French studies have previously reported that a non-negligible proportion of patients with IPF were subsequently diagnosed as having AAV. [3–5] Since both IPF and AAV typically affect elderly adults, and since the cost of serologic testing for ANCA is relatively low, we believe that it should be performed even in the absence of any extra-pulmonary signs of vasculitis at the time of initial presentation. This could help the differential diagnosis procedure, and may also increase therapeutic options in a disease that is all too often rapidly fatal. We take the opportunity of a case presentation to review the literature on this subject and to discuss this opinion.

## Case presentation

**Fig. 1:**
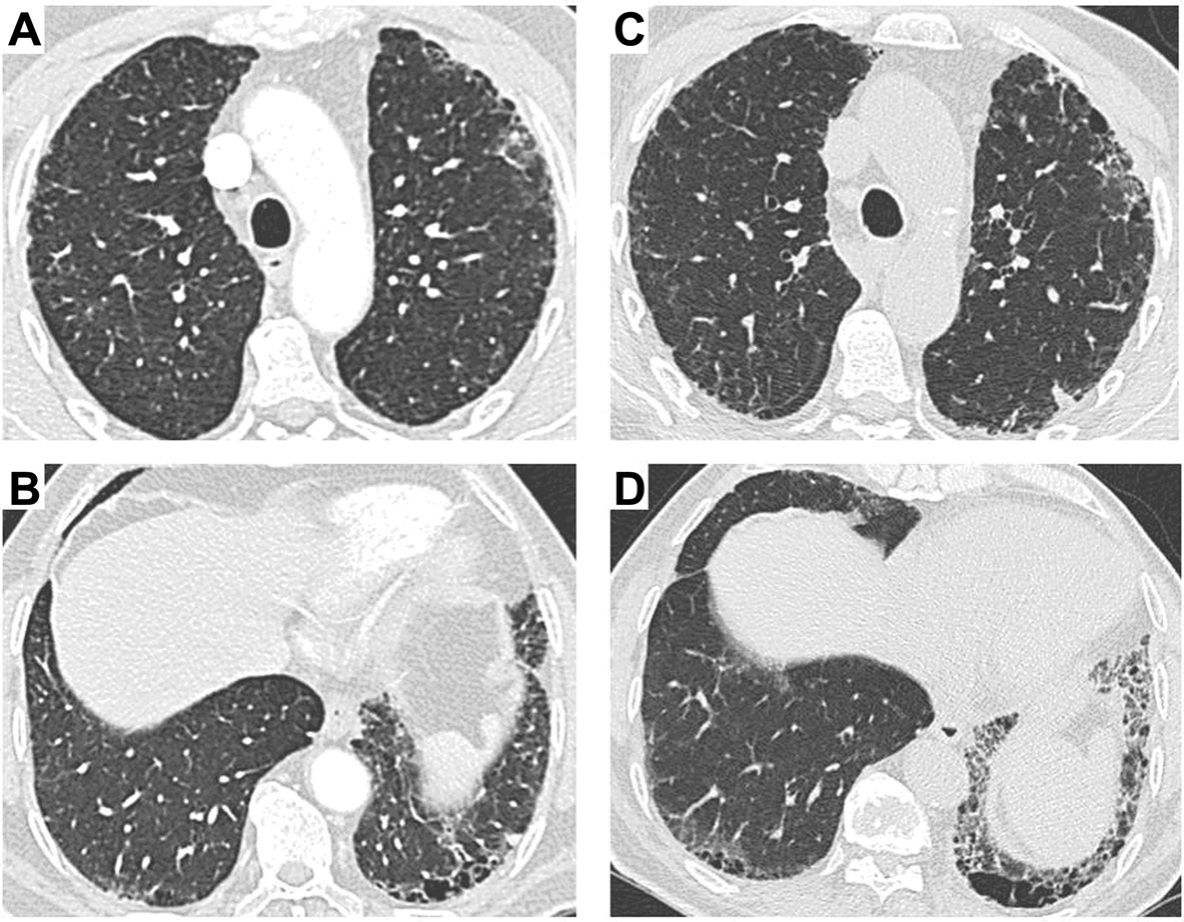
High-resolution computed tomography scan findings in year 2009 (panels **a** and **b**) and 2013 (panels **c** and **d**). Both examinations revealed sub-pleural reticulation, honeycombing, traction bronchiectasis. Lesions predominated in the lower part of the lungs, and had progressed between 2009 and 2013

**Fig. 2:**
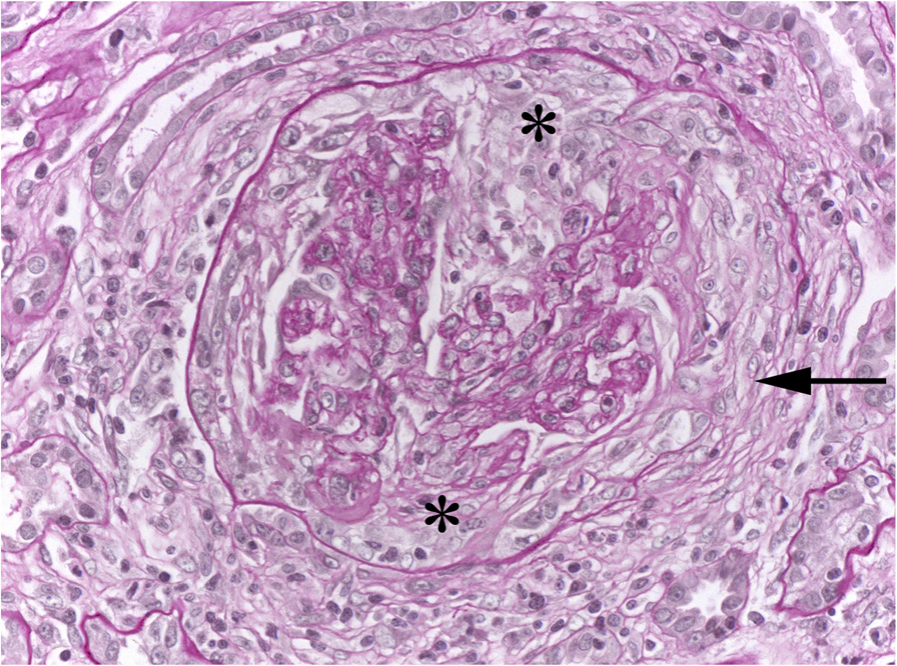
Renal biopsy findings. The glomerulus shows active segmental necrotizing destructive crescent (asterisk) with destruction of Bowman’s capsule (arrow). (Periodic Acid Schiff, original magnification x250)

A 71-year old man, suffering from chronic cough, was diagnosed with idiopathic lung fibrosis in 2010. He had a former smoking history of 20 pack years (he stopped smoking in 2001), but no previous relevant environmental exposure. Except for bilateral basal crackles, the physical examination was unremarkable. A computerized tomography [CT] scan showed reticular opacities, honeycombing, and some traction bronchiectasis; fibrotic lesions were predominant in the lower parts of lungs. No ground-glass opacities were found [Fig. 1]. Pulmonary test function was normal, except for the corrected carbon monoxide [CO] diffusing capacity, which was slightly reduced (60 %). A six-minute walking test was normal. Cellular analyses of a bronchoalveolar lavage [BAL] found 240,000 cells/mm^3^ consisting of 80 % macrophages (91 % siderophages), 9 % neutrophils (N < 1 %), and 11 % lymphocytes (N < 10 %). Serum creatinine was within the normal range (0.8 mg/dL), and hemoglobin concentration was 14.5 g/dL. Urine sediment was not specifically analyzed, but a urinary dipstick had found mild microscopic hematuria (1+) and no albuminuria. Serum was not screened for ANCA. A final diagnosis of IPF was made. This patient was lost to follow-up until 2013, when he presented to the pulmonary ward because of dyspnea on exertion. On that same day, his serum creatinine was still normal (1 mg/dL). He was scheduled for lung function tests 4 months later. At that time, his general condition had deteriorated and he presented with elevated serum creatinine (6.1 mg/dL) and acute anemia (9 g/dL versus 13 g/dL 4 months earlier) in the absence of hemoptysis. His uncorrected CO diffusing capacity was even lower than in 2010 (38 %), while his corrected CO diffusing capacity had increased slightly (66 %). A CT-scan showed progression of fibrotic lesions and again no ground-glass opacities [Fig. 1]. A urinary dipstick found albuminuria (>3 g/L) and microscopic hematuria (3+). He was then referred to our renal intensive care unit. Serum fibrinogen was mildly increased (4.76 gr/L), and C3 and C4 were in the normal range (0.97 and 0.26 gr/L, respectively). A high titer of anti-myeloperoxidase ANCA was found, and a renal biopsy revealed necrotizing crescentic pauci-immune glomerulonephritis [Fig. 2]. Glomerular lesions of different ages and the coexistence of active necrotizing crescentic lesions (50 %) and sclerosing segmental or diffuse glomerular lesions (33 %) were observed, suggesting unrecognized flares of glomerulonephritis in the past. The lower cortex was infiltrated by mononuclear cells and active tubulitis was focally observed.

The 2010 serum was retrospectively screened for the presence of ANCA, which were found at a low level (1:40); with a perinuclear pattern visible by immunofluorescence. In contrast to the most recent serum test, neither MPO nor PR3 specificity was found. Plasmapheresis, methyl-prednisolone, and cyclophosphamide were promptly administered. Renal and pulmonary functions eventually improved. The serum creatinine, three months post admission, was 2.4 mg/dL. A maintenance therapy using rituximab was decided. Eighteen months later, both his renal and respiratory functions were stable: urinary sediment was inactive, proteinuria was low (20 mg/mmol creatinine), serum creatinine was 2.7 mg/dL (estimated glomerular filtration rate 23 ml/min) and his pulmonary forced vital capacity was 2930 mL (76 %). He had not experienced any other flare, and ANCA remain undetectable, both by immunofluorescence and ELISA.

## Discussion

In their historic article suggesting that ANCA play a pathogenic role on AAV, Jennette et al. reported that alveolar interstitial fibrosis was frequently observed in patients with lung manifestations of vasculitis [6]. One patient in particular is said to have undergone repeated lung biopsies, the first one showing alveolar capillaritis alone but the second only interstitial fibrosis [6]. The retrospective analysis of the case of our patient, including his serum tests, makes a diagnosis of occult flares of alveolar capillaritis and glomerulonephritis rather obvious. However, the current guidelines regarding the diagnosis of IPF state that performing a BAL cellular analyses in the setting of IPF is at the discretion of the treating physician and screening for ANCA is not even discussed [2].

The largest study so far to measure the prevalence of ANCA in patients with IPF was published by Ando et al. [7]. At the time of diagnosis 3/61 patients (4.9 %) were found to have circulating MPO-ANCA, and this rose to 9/61 (14.8 %) during follow up. Two of these 9 patients went on to develop crescentic glomerulonephritis and, thus, had a robust final diagnosis of vasculitis. Patients with MPO-ANCA were more frequently found to have rheumatoid factor, a lower mean CO diffusing capacity, and a lower macrophage count on BAL cellular analyses. Histopathology of 7/9 patients with MPO-ANCA associated IPF revealed a typical pattern of interstitial pneumonia in all cases. This study, as well as that by Hervier et al. [5] which included 12 such patients, reported an increased number of eosinophils either in the blood [5] or in the bronchoalveolar fluid [7]. With respect to patient outcome, comparing ANCA+ and ANCA- IPF on a prospective basis did not reveal any significant differences [4, 7] and, in particular, survival rates were similar [7]. In line with this, a retrospective study reported on 53 IPF patients who were secondarily tested for the presence of proteinase 3 [PR3] and myeloperoxidase [MPO] ANCA. This showed that 19/53 patients (35.8 %) were positive for ANCA, including 17 with MPO and 2 with PR3 specificity. Four patients out of 19 had a definite diagnosis of micropolyangitis. There were no major differences in clinical or biological features between ANCA+ and ANCA- patients, and pulmonary function tests, BAL, and radiological findings all yielded comparable results. As expected though, immunosuppressive treatment had been more frequently used in the ANCA-positive patients, and a trend toward a higher rate of improvement was observed in the ANCA-positive group (83.3 % vs 54.2 %, *p =* 0.089).

For reasons that are not immediately obvious, the combination of IPF with AAV is still reported in the literature as an association of two distinct diseases with potential pathophysiological links, certainly, but never as manifestations of one and the same disorder, namely vasculitis, leading to a fibrosing injury/repair response in the lung. The lung is frequently involved in micropolyangitis, although mainly through capillaritis and alveolar hemorrhage. ANCA-associated pulmonary fibrosis typically precedes AAV [8] by several months and sometimes by several years, and so it is argued that a sequence of events in which IPF is accompanied by ANCA, but with no patent alveolar capillaritis, and which is followed by vasculitis suggests that the ANCA is not involved in the vasculitis *per se*, but rather in IPF via some unknown mechanism(s). First, what are the odds that a patient will be diagnosed with an *idiopathic* disease once he/she has been diagnosed as having systemic vasculitis ? Second, ANCA were first described decades ago and their pathophysiological role has been extensively studied, yet we do not know that they have been involved in any case of isolated abnormal matrix remodeling *without* vasculitis, whether in humans or in animal models. Experimental models have suggested that ANCA mediate vasculitic lesions via neutrophil activation, which with different mediators, such as lipopolysaccharide and tumor necrosis alpha [9], will harm tissues. Obviously enough, tissue injury will subsequently induce the activation of fibroblasts and epithelial cells and cause fibrosis, but the *primum movens* here is vasculitis, whether patent or occult. Taking into account the fact that cases of occult flares of vasculitis, whether in the lung, the kidney or other organs, are known to occur, and the fact that AAV evolves in flares, we think that the time has come to include the detection of ANCA as a reason to prompt further discussion of chronic and indolent vasculitis in the differential diagnosis of a patient presenting with IPF. The prognosis of this combination is particularly bad [8]. This implies testing for ANCA on a systematic basis and, if they are found to be present, to perform BAL cellular analyses to look for alveolar hemorrhage and a urinary dipstick to look for microscopic hematuria. Indeed, non-specific CT findings and a pattern of usual interstitial pneumonitis (UIP) without patent signs of alveolar hemorrhage does not rule out a diagnosis of micropolyangitis.

The PANTHER trial has firmly demonstrated that a combination of prednisone and azathioprine does not afford any benefit in patients with IPF in general (it was even shown to increase mortality and acute exacerbation of the disease) [10]. However, evidence from the literature is scarce about the real efficacy of immunosuppressive drugs in ANCA-associated pulmonary fibrosis. In addition, immunosuppressive drugs have potent anti-inflammatory properties, but do not act on matrix remodeling, hence it is counter-intuitive to think that they would display much efficacy against an inflammatory disease discovered at a late/fibrotic stage, in contrast with pirfenidone, for example [11].

Taking all these factors into consideration, we believe that patients presenting with IPF should be screened for ANCA, because pulmonary fibrosis could be the end-result of repeated mild flare(s) of lung vasculitis causing occult alveolar hemorrhage. In the kidney field, it is widely accepted that glomerulosclerosis may reflect previous flares of vasculitis of the glomerular capillary, with the alveolar capillaries probably sharing the same pattern of injury/repair response and scarring. The pathophysiological link between indolent vasculitis and fibrogenesis is the subject of speculation, but local inflammation and the release of heme proteins into the alveolar chamber could, for instance, induce oxidative damage and destabilize lung homeostasis. Two important observations were made in a recent study assessing the various histopathological features of AAV with renal involvement : 1) 20.3 % of patients with MPO-AAV were found to display exclusively chronic/sclerosing lesions at the renal biopsy and 2) half the patients presented with lesions of differing ages, whereas the diagnosis of AAV was recent (1.6 month in the MPO-AAV group) [12].

For those patients with ANCA associated IPF, we speculate that they should be under close scrutiny in order to capture infra-clinical flares of a vasculitis, and treat them according to the current guidelines on AAV. In particular, we recommend that they undergo at least one BAL to detect broncho-alveolar hemorrhage, and urinary dipsticks on a monthly basis. Microscopic hematuria and proteinuria are sensitive biomarkers of glomerular injury, and would rapidly lead to a renal biopsy and correct the diagnostic. Outside flares, there is no evidence-based argument to add anti-fibrotic drugs if a diagnosis of AAV is strongly suspected or even established. It should also be born in mind that the risk of cancer, increased by immunosuppressants, could theoretically be further increased by a stromal depletion [13]. In the case we present, the use of plasmapheresis at the time of extracapillary glomerulonephritis, and of rituximab as maintenance, were decided on the basis of recent randomized trials (MEPEX [14], and MAINRITSAN [15], respectively).

## Conclusion

In conclusion, we suggest moving from a radiological and histopathological classification of this disease to a more molecular and mechanistic approach. A significant proportion of patients presenting with pulmonary fibrosis who test positive for ANCA should be scrutinized closely and, for them, vasculitis is a disease to look out for during follow-up. If respiratory or extra-respiratory signs of vasculitis are found, this would undoubtedly increase therapeutic options in a disease which, at present, is rapidly fatal.

## Ethics/consent

Written informed consent was obtained from the patient for publication of this case report and of accompanying images.

## Abbreviations

AAV: ANCA associated vasculitis
ANCA: Anti-neutrophil cytoplasmic antibodies
BAL: Broncho-alveolar lavage
CO: Carbon monoxide
CT: Computerized tomography
IPF: Idiopathic pulmonary fibrosis
MPO: Myeloperoxidase
PR3: Proteinase 3
